# Loss of *EMI1* compromises chromosome stability and is associated with cellular transformation in colonic epithelial cell contexts

**DOI:** 10.1038/s41416-024-02855-9

**Published:** 2024-10-02

**Authors:** Rubi Campos Gudiño, Nicole M. Neudorf, Demi Andromidas, Zelda Lichtensztejn, Kirk J. McManus

**Affiliations:** 1 Paul Albrechtsen Research Institute, CancerCare Manitoba, Winnipeg, MB Canada; 2 Department of Biochemistry and Medical Genetics, Rady Faculty of Health Sciences, University of Manitoba, Winnipeg, MB Canada

**Keywords:** Cancer genetics, Colorectal cancer, Genetics research

## Abstract

**Background:**

Colorectal cancer (CRC) is still a leading cause of cancer deaths worldwide. Thus, identifying the aberrant genes and proteins underlying disease pathogenesis is critical to improve early detection methods and develop novel therapeutic strategies. Chromosome instability (CIN), or ongoing changes in chromosome complements, is a predominant form of genome instability. It is a driver of genetic heterogeneity found in ~85% of CRCs. Although CIN contributes to CRC pathogenesis, the molecular determinants underlying CIN remain poorly understood. Recently, *EMI1*, an F-box protein, was identified as a candidate CIN gene. In this study, we sought to determine the impact reduced *EMI1* expression has on CIN and cellular transformation.

**Methods:**

Coupling siRNA-based silencing and CRISPR/Cas9 knockout clones with quantitative imaging microscopy we evaluated the impact reduced *EMI1* expression has on CIN and cellular transformation in four colonic epithelial cell contexts.

**Results:**

Quantitative imaging microscopy data revealed that reduced *EMI1* expression induces increases in CIN phenotypes in both transient (siRNA) and constitutive (CRISPR/Cas9) cell models that are associated with increases in DNA damage and cellular transformation phenotypes in long-term studies.

**Conclusions:**

This study determined that reduced *EMI1* expression induces CIN and promotes cellular transformation, which is consistent with a role in early CRC development.

## Introduction

Colorectal cancer (CRC) is the third most diagnosed and second most lethal cancer worldwide. Each year, ~2 million individuals are newly diagnosed, while an additional ~900,000 individuals succumb to the disease, which accounts for ~10% of all cancer diagnoses and deaths, respectively [1, 2]. As such, understanding the molecular determinants (i.e., aberrant genes, proteins and pathways) driving disease development is critical to advance our understanding of early disease development and to exploit this information to identify early biomarkers of disease and/or develop innovative therapeutic strategies to ultimately improve the lives and outcomes of those diagnosed with CRC. In this regard, chromosome instability (CIN) is a prevalent form of genome instability that is associated with ~85% of all CRC cases, suggesting it may be a pathogenic event in CRC development [3].

CIN is defined as an increase in the rate at which whole chromosomes or large chromosome fragments are gained and/or lost and is a driver of genetic and cellular heterogeneity [3, 4]. Conceptually, gains and/or losses of chromosomes impact gene copy numbers and expression patterns such that gains may promote the overexpression of oncogenes, while losses may reduce tumour suppressor gene expression [5–7]. Overall, CIN is a dynamic phenotype that drives ongoing karyotypic evolution. Accordingly, CIN is proposed to respond to selective pressures to promote growth, proliferation, and cell survival [3, 8, 9] that are associated with cellular transformation [5–7, 10, 11], tumour evolution [12], metastases [13], the acquisition of drug resistance, and consequently, poor patient prognosis [14]. Despite all of these associations, the molecular determinants underlying CIN remain poorly understood.

We previously determined that reduced expression of core members of the SCF complex (SKP1 [S-Phase Kinase Associated Protein 1]; CUL1 [Cullin 1]; F-box protein) induces CIN and cellular transformation in colorectal and ovarian cancer contexts, supporting the possibility that they are aberrant aetiological events underlying early disease development [15–17]. The SCF complex is an E3 ubiquitin ligase that polyubiquitinates protein substrates to mark them for degradation via the 26S proteasome [18, 19]. It is comprised of three invariable core members, SKP1, CUL1 and RBX1 (RING-Box Protein 1), and one of 69 variable F-box proteins, like EMI1 (Early Mitotic Inhibitor-1; also known as FBXO5) that impart substrate specificity to the complex [20, 21]. Our previous findings suggest that normal SCF complex function is required to maintain genome integrity, chromosome stability and prevent oncogenesis [9, 15, 16, 22]. Although these initial studies only focused on the three core members, they also suggest that aberrant expression of F-box proteins, such as *EMI1*, may also induce CIN, promote cellular transformation and contribute to early disease development.

A comprehensive siRNA-based screen of all 69 F-box proteins performed in HCT116 identified *EMI1* as the top candidate CIN gene, as its silencing induced the greatest increases in nuclear areas [23]. *EMI1* was originally identified in a yeast two-hybrid screen designed to identify novel F-box proteins [24] and is an essential gene [25, 26]. EMI1 binds target substrates and is subsequently recruited to form a fully functional SCF complex (SCF^EMI1^) to target substrates like RAD51 (Radiation Deficient Recombinase 51) for proteolytic degradation [27]. Although RAD51 is the only SCF^EMI1^ substrate target identified to date, it is expected that EMI1, like other F-box proteins, targets tens of proteins for proteolytic degradation [28–30]. While *EMI1* is traditionally described as an oncogene, given its overexpression induces CIN and tumourigenesis [31], there is a lack of information regarding its potential as a tumour suppressor gene as it is frequently lost in many cancers [32], and reduced expression is predicted to underlie aberrant increases in oncoproteins that may promote CIN and cancer development. Accordingly, an in-depth evaluation of the impact reduced *EMI1* expression has on CIN and cellular transformation is highly warranted.

To determine the prevalence and potential clinical impact of *EMI1* copy number losses in cancer, publicly available TCGA (The Cancer Genome Atlas) data from 10 common cancer types [33] were queried using cBioPortal [34, 35]. TCGA data reveal that *EMI1* copy number losses (i.e., shallow deletions) occur frequently and correspond with increases in both the fraction of the genome altered and aneuploidy scores in CRC, suggesting reduced expression may induce CIN. Moreover, reduced expression is associated with significantly worse disease-specific and progression-free survival for CRC patients relative to those with normal (diploid) copy number status, suggesting it may have pathogenic implications. To functionally determine the impact reduced *EMI1* expression has in CRC, *EMI1* silencing and quantitative imaging microscopy (QuantIM) were performed in four karyotypically stable, colonic epithelial cell lines. Notably, reduced expression in all four lines induced significant increases in CIN phenotypes that included changes in nuclear areas, micronucleus formation and aberrant chromosome numbers. To determine the long-term impact reduced *EMI1* expression has on CIN, clinically relevant, heterozygous knockout clones (*EMI1*^+/^^−^) were generated and assessed every 2 weeks over a 10-week period. In agreement with the silencing experiments, heterozygous loss of *EMI1* and reduced expression induced ongoing and dynamic changes in CIN phenotypes that coincide with increases in DNA double-strand breaks (DSBs) along with increases in proliferation rates and anchorage-independent growth that are consistent with reduced expression inducing cellular transformation. Collectively, our data show that reduced *EMI1* expression induces CIN that promotes cellular transformation, which supports a potential pathogenic role for heterozygous loss of *EMI1* in early CRC development.

## Methods

### *EMI1* clinical assessments

Publicly available gene copy number, mRNA expression and clinical data were extracted from the TCGA Pan-Cancer Atlas dataset [33] for 10 common cancer types (breast, cervical, CRC, glioblastoma, head & neck, liver, lung, ovarian, prostate and uterine) using cBioPortal [34, 35] and as detailed elsewhere [36]. *EMI1* mRNA expression data from samples with deep and shallow deletions were imported onto Prism v9 (GraphPad, San Diego, CA, USA), where cases with shallow deletions and diploid status were statistically compared using a Mann–Whitney (MW) test. The fraction of the genome altered and aneuploidy score data from CRC patients were also imported onto Prism, where statistical comparisons (MW tests) were performed between cases harbouring shallow deletions relative to diploid controls. Clinical outcomes data, namely disease-specific and progression-free survival, were extracted from TCGA data [33] and stratified based on *EMI1* copy number status—shallow deletion versus diploid cases. Kaplan-Meier (KM) survival curves were generated and statistically compared using log-rank tests with a *p*-value < 0.05 being considered statistically significant.

### Cell lines and culture

Four karyotypically stable, diploid/near-diploid colonic epithelial cell lines were employed. HCT116 (male, modal chromosome number = 45) and SW48 (female, modal chromosome number = 47; Fig. S1) are malignant lines purchased from American Type Culture Collection (ATCC, Rockville, MD, USA), while 1CT and its derivative, A1309 (male, modal chromosome number = 46) are non-malignant and were generously provided by Dr. Jerry W. Shay (University of Texas Southwestern, Dallas, TX, USA) [37, 38]. All lines are karyotypically stable, and HCT116, 1CT and A1309 have previously been employed in similar CIN-based studies [17, 23, 36, 39], while SW48 was included as a karyotypically stable, female cell line as determined by spectral karyotyping and mitotic chromosome spread analyses (Fig. S1, Table S1). 1CT and A1309 are immortalised with human telomerase reverse transcriptase (hTERT) and cyclin-dependent kinase 4 (CDK4); however, A1309 harbours reduced *Tumour Protein P53* (*TP53)* expression, produces a mutant form of Kirsten Rat Sarcoma Proto-Oncogene (KRAS^G12V^) and expresses *Adenomatous Polyposis Coli* (*APC*) truncated at amino acid residue 1309 [37, 38]. HCT116 cells were cultured in McCoy’s 5A medium (Cytiva HyClone, Vancouver, BC, Canada) supplemented with 10% fetal bovine serum (Sigma-Aldrich, St. Louis, MO, USA), while SW48 were cultured in Leibovitz’s L-15 medium (Gibco, Grand Island, NY, USA) supplemented with 10% fetal bovine serum (Cytiva HyClone). 1CT and A1309 were cultured in Dulbecco’s Modified Eagle Medium with High Glucose/Medium 199 (Cytiva HyClone) and supplemented with 10% cosmic calf serum (Cytiva HyClone). All cell lines were authenticated based on protein expression and/or karyotypic analyses [36]. All cell lines tested negative for mycoplasma contamination. HCT116 cells were grown in a humidified incubator at 37 °C with 5% CO_2_. SW48 were grown in a humidified incubator at 37 °C without CO_2_ supplementation. Lastly, 1CT and A1309 were grown in low oxygen chambers filled with 2% O_2,_ 7% CO_2_, and 91% N_2_ at 37 °C [37, 38].

### *EMI1* silencing and western blot

*EMI1* silencing was performed using RNAiMAX (Life Technologies, Canada) and ON-TARGETplus siRNA duplexes (Horizon Discoveries Biosciences Ltd., Cambridge, UK). Briefly, four individual siRNA duplexes (siEMI1-1, -2, -3, -4) targeting unique coding regions of the *EMI1* mRNA or a pool (siEMI1-Pool [siEMI1-P]) comprised of an equimolar combination of the four individual siRNAs were employed, as well as non-targeting siRNA control (siControl). Silencing efficiencies were evaluated by western blots three (HCT116) or four days (SW48, 1CT, A1309) post-transfection (i.e., similar population doublings) [40], using the antibodies and dilutions specified in Table S2. Semi-quantitative analyses were employed to obtain relative protein expression of EMI1 and RAD51 as detailed elsewhere [15, 23].

### Single-cell quantitative imaging microscopy (QuantIM) and CIN analyses

QuantIM approaches were employed to assess changes in CIN phenotypes, including nuclear areas and micronucleus formation, as detailed elsewhere [9, 41]. Briefly, cells were seeded into 96-well plates, silenced in sextuplet and allowed to grow for 3–4 days, after which cells were fixed (4% paraformaldehyde) and the DNA was counterstained (Hoechst 33342). To perform quantitative analyses, 3 × 3 or 4 × 4 matrices of non-overlapping images were acquired from each well using a Cytation 3 Cell Imaging Multi-Mode Reader (BioTek, Winooski, VT, USA) equipped with a 20× objective. Nuclear areas and micronucleus formation from a minimum of 300 nuclei/condition were automatically quantified using Gen5 software (BioTek), as detailed previously [9, 41]. All quantitative data were imported into Prism, where descriptive statistics and non-parametric tests were performed, including two-sample Kolmogorov-Smirnov (KS) tests comparing cumulative nuclear area distribution frequencies and MW tests assessing differences in micronucleus formation frequencies. Additionally, analysis of variance (ANOVA) and Tukey-posts tests were conducted on all pair-wise combinations for the non-targeting control (NT-Control) clone at the different passages (p0-p20). For all statistical tests, a *p*-value < 0.05 is considered statistically significant. Experiments were performed in triplicate, and all graphs were generated in Prism, with figures assembled in Photoshop 2024 (Adobe, San Jose, CA, USA).

### Mitotic chromosome spreads generation and enumeration

Mitotic chromosome spreads were generated as detailed elsewhere [40, 42] with a minimum of 100 spreads enumerated per condition. All experiments were performed in triplicate except for the temporal *EMI1*^*+/*^^−^ clone studies, in which each clone was assessed once at each time point. Two-sample KS tests were employed to identify statistically significant differences in the chromosome number cumulative distribution frequencies of *EMI1* silenced cells relative to siControl and the *EMI1*^*+/*^^−^ clones relative to NT-Control clone.

### CRISPR/Cas9 approaches to generate *EMI1* knockout clones in A1309 cells

*EMI1* knockout clones were generated using a two-step CRISPR/Cas9 approach in A1309 cells with *EMI1*-targeting and non-targeting control synthetic guide RNAs (sgRNAs) according to the manufacturer (Sigma-Aldrich) and as detailed previously [15]. Briefly, cells were transduced with lentivirus particles containing two unique *EMI1* sgRNAs (Table S3) or a non-targeting sgRNA control (NT-Control; Table S3) that co-express blue fluorescent protein (BFP). BFP+ cells were isolated using fluorescence-activated cell sorting (FACS) and subsequently transfected with a plasmid that co-expresses Cas9 and green fluorescent protein (GFP). BFP+/GFP+ cells were isolated by FACS, and individual clones were obtained using serial dilution. Putative *EMI1* knockout clones were identified by western blot (reduced EMI1 abundance), with allele-specific edits identified with DNA sequencing (Génome Quebec, Montreal, QC, Canada).

### Proliferation assay

Proliferation rates were determined using CellTiter-Glo according to the manufacturer (Promega, Madison, WI, USA). Briefly, *EMI1*^*+/*^^−^ and NT-Control clones were seeded in four 96-well plates at 100 cells/well in sextuplet. Cells were grown for up to 6 days and analysed on days 3, 4, 5 and 6, with a standard curve generated using wild-type untreated cells seeded at pre-defined densities (0; 500; 1000; 2000; 4000; 8000; 12,000; 16,000 cells/well). Luminescence was measured using a Cytation 3 with Gen5 software, and proliferation rates (i.e., doubling times) were calculated for early (p0) and late (p20) passage cells using the following formula:

$${doubling\; time}=\frac{[72* \log \left(2\right)]}{[\log \left({mean\; cell\; count\; at}\;144\,h\right)-\log \left({mean\; cell\; count\; at}\;72\,h\right)]}$$

### Soft-agar colony formation assay

3D colony formation assays were performed as described elsewhere [42, 43] and were performed on early (p0) and late (p20) passage cells. Briefly, clones were seeded (20,000 cells/well) in 0.4% agar into a 6-well plate containing a base layer of 0.6% agar. Cells were supplemented with media and replaced every week for 4 weeks, at which point cells were fixed (4% paraformaldehyde), stained (0.005% crystal violet) and imaged using a Cytation 3 equipped with a 4× objective. Gen5 was employed to enumerate and measure colonies, with individual colonies being operationally defined as those that meet a minimum diameter of ≥100 µm and an area >0.01 mm^2^, which equates to ~50 cells.

### QuantIM assessment of DNA DSBs

Asynchronous *EMI1*^+/^^−^ and NT-Control clones were fixed, permeabilized, immunofluorescently labelled with anti-γ-H2AX (Abcam; ab26350; 1:200) and anti-53BP1 (Abcam; ab175933; 1:200) antibodies and subjected to QuantIM as detailed elsewhere [44]. Briefly, each channel was independently optimised using a positive control (Bleomycin; 1μg/ml; 2 h) and maintained constant throughout the entire acquisition phase (Zeiss Axio Imager 2; 40 × objective). Image analyses quantified the number of γ-H2AX foci and the 53BP1 total signal intensities for each interphase nucleus imaged, with a minimum of 200 nuclei imaged per condition. The total number of γ-H2AX foci was statistically compared to controls (DMSO-treated or untreated NT-Control clone) using one-sided Mann–Whitney tests, while mean 53BP1 total signal intensities were compared using one-sided Student’s *t*-tests in Prism, with *p*-values < 0.05 considered statistically significant. Descriptive statistics (e.g., *N*, mean, quartiles) were generated in Prism with graphs exported into Photoshop, where figures were assembled.

## Results

### Heterozygous loss of *EMI1* is associated with genome instability and poor patient outcomes in CRC

**Fig. 1:**
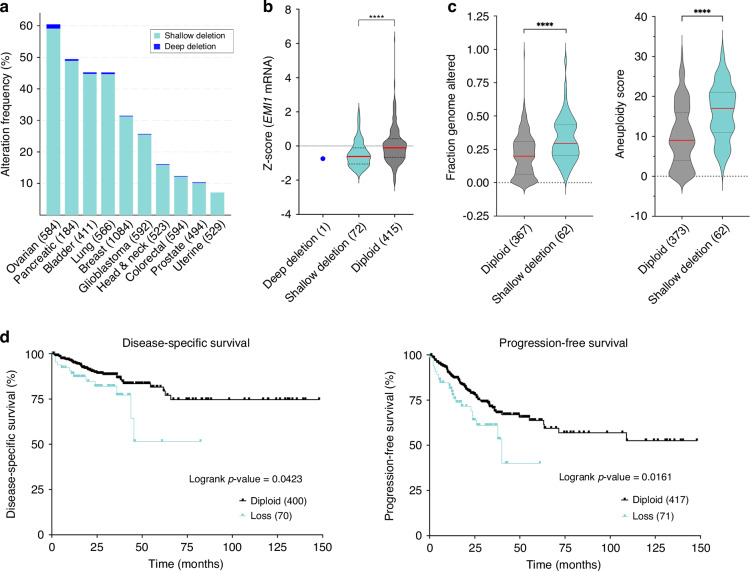
*EMI1* copy number losses are frequent in cancer and are associated with genome instability and worse patient outcomes in CRC. **a** Bar graph presenting the frequency of *EMI1* copy number losses, including deep (homozygous) and shallow (heterozygous) deletions in ten cancer types (total cases) [33–35]. Note that ~12% of CRC cases exhibit *EMI1* copy number losses. **b** Violin plot reveals *EMI1* copy number losses correspond with a significant reduction in mRNA expression in CRC cases relative to diploid counterparts (MW test; ****p-value < 0.0001). Red bar identifies median, while dashed lines identify interquartile range. **c** CRC patients with *EMI1* shallow deletions exhibit significant increases in the fraction of the genome altered (left) and aneuploidy scores (right) relative to diploid counterparts (MW test; ****p-value < 0.0001) [45, 46]. **d** Kaplan-Meier curves reveal significantly worse disease-specific (left) and progression-free survival (right) for CRC patients with *EMI1* shallow deletions (loss) relative to diploid cases [33–35].

To determine the clinical impact *EMI1* copy number losses and reduced expression may have in cancer, bioinformatic analyses were performed using publicly available TCGA data [33–35]. First, the prevalence of *EMI1* copy number losses, specifically deep (homozygous) and shallow (heterozygous) deletions, were assessed in 10 common cancer types. As shown in Fig. 1a, copy number losses occur in all 10 cancer types and range from 7% in uterine cancers to 59% in ovarian cancers. While shallow deletions are only present in ~12% of CRC cases, this equates to ~240,000 new diagnoses annually throughout the world. Furthermore, shallow deletions in CRC correspond with significant decreases in mRNA expression (Fig. 1b; protein abundance is not available), which are associated with increases in genome instability, namely the fraction of the genome altered and aneuploidy scores (i.e., sum of total altered chromosome arms; Fig. 1c) [34, 35, 45, 46]. Moreover, CRC patients with *EMI1* shallow deletions have significantly worse clinical outcomes relative to diploid counterparts, including both disease-specific and progression-free survival (Fig. 1d). Collectively, these data support the possibility that reduced *EMI1* expression induces CIN that promotes CRC pathogenesis and contributes to worse patient outcomes.

### *EMI1* silencing induces increases CIN in a male CRC cell line

**Fig. 2:**
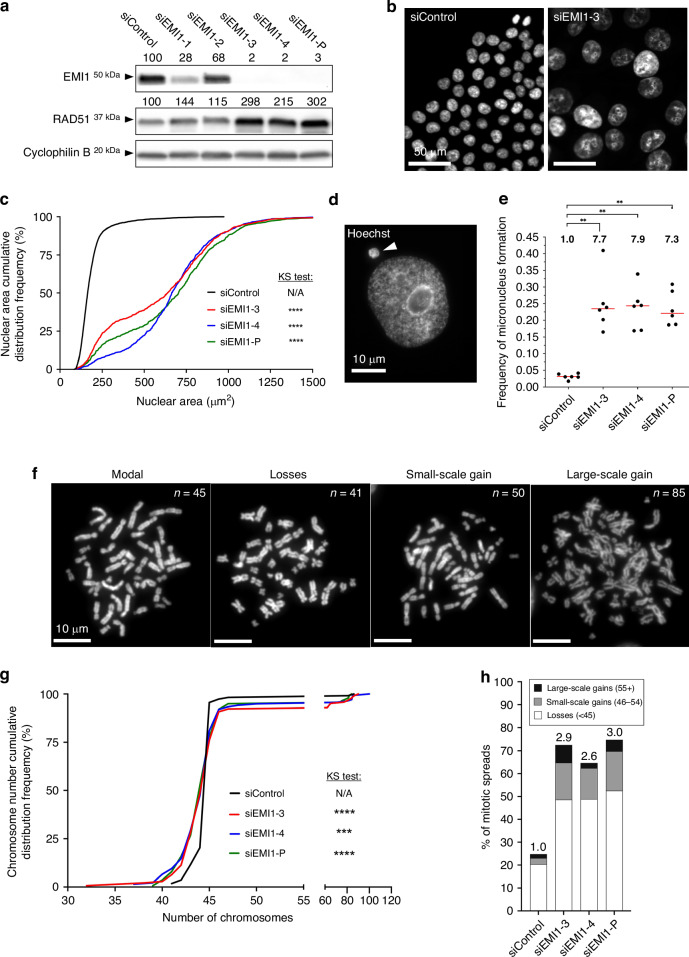
Transient *EMI1* silencing induces significant increases in CIN phenotypes in HCT116 cells. **a** Semi-quantitative western blots depicting the silencing efficiencies of four individual siRNAs targeting *EMI1* (si*EMI1*-1, -2, -3, -4) and a pooled condition (si*EMI1*-Pool [si*EMI1*-P]). EMI1 and RAD51 abundance are normalised to Cyclophilin B (loading control) and presented relative to siControl (set to 100%) (*n* = 3). **b** Low-resolution images of Hoechst-counterstained nuclei showing visual increases in nuclear areas following *EMI1* silencing. **c** Cumulative nuclear area distribution frequency graph reveals significant increases in nuclear areas following *EMI1* silencing (two-sample KS test; N/A not applicable; ****p-value < 0.0001; *n* = 3; > 300 nuclei analysed/condition). **d** High-resolution image of a micronucleus (arrowhead). **e** Dot plot reveals significant increases in the frequency of micronuclei following *EMI1* silencing. Median values indicated by red bars, while fold increase relative to siControl are presented above each column (MW test; **p-value < 0.01; *n* = 3; 6 wells analysed/condition). **f** Representative high-resolution images of mitotic chromosome spreads displaying the modal number of 45 chromosomes, chromosome losses (< 45 chromosomes), small-scale gains (46–54 chromosomes), and large-scale gains (> 54 chromosomes). Chromosome numbers (*n*) are indicated at the top right of each image. **g** Chromosome number cumulative distribution frequency graph showing statistically significant changes in distributions following *EMI1* silencing relative to siControl (two-sample KS test; N/A, not applicable; ***p-value < 0.001; ****p-value < 0.0001; *n* = 3; ≥ 100 spreads analysed/condition). **h** Bar graph presenting the frequencies of aberrant chromosome spreads following *EMI1* silencing, with the fold increase relative to siControl indicated above each bar (*n* = 3; ≥ 100 spreads analysed/condition).

To assess the impact reduced *EMI1* expression has on CIN, we coupled transient siRNA-based silencing and QuantIM in HCT116, a male, karyotypically stable malignant CRC cell line used extensively in previous CIN studies [17, 23, 36, 39]. However, prior to performing QuantIM assays, we first established the silencing efficiencies of four individual siRNA duplexes (si*EMI1*-1, -2, -3, -4) and a pooled siRNA condition (si*EMI1*-Pool [si*EMI1*-P]). As shown in Fig. 2a, semi-quantitative western blots identified si*EMI1*-3 and -4 as the most efficient duplexes, which, together with si*EMI1*-P, reduced EMI1 abundance to ≤3% of siControl levels and, thus, were selected for all downstream analyses. In agreement with RAD51 being an established target of the SCF^EMI1^ complex [27], *EMI1* silencing induced a 2- to 3-fold increase in RAD51 abundance (Fig. 2a) confirming an on target and functional consequence of reducing *EMI1* expression.

To determine the impact reduced *EMI1* expression has on CIN in HCT116 cells, we employed QuantIM to assess CIN phenotypes, or more specifically, changes in nuclear areas, micronucleus formation and alternations in chromosome numbers. Briefly, changes in nuclear areas typically correspond with large changes in chromosome complements (i.e., ploidy), while micronuclei are small extranuclear DNA-containing bodies that often arise due to chromosome missegregation events during mitosis and are hallmarks of CIN [47–49]. In general, *EMI1* silencing corresponded to visual increases in nuclear areas relative to siControl (Fig. 2b) that quantitative analyses revealed were statistically significant (Fig. 2c, Table S4). *EMI1* silencing also induced significant, ~7-fold increases in micronucleus formation relative to siControl (Fig. 2d, e; Table S5). Finally, chromosomes were manually enumerated from mitotic chromosome spreads and all spreads harbouring aberrant (i.e., non-modal) chromosome numbers were classified into one of three categories: 1) losses in which one or more chromosomes were lost; 2) small-scale gains involving nine or fewer chromosomes; or 3) large-scale gains involving 10 or more chromosomes (Fig. 2f). Recall that HCT116 has a modal chromosome number of 45, which is operationally defined as the ‘normal’ state. As shown in Fig. 2g, *EMI1* silencing induced significant changes in chromosome distributions relative to siControl (Table S6). Moreover, these changes corresponded with a 2.6- to 3.0-fold increase in the frequency of aberrant spreads that includes overall increases within each aberrant category (Fig. 2h). Further scrutiny of the images also revealed evidence of endoreduplication, or subsequent rounds of DNA replication (S-phase) in the absence of cytokinesis [50, 51], with ~33% of the spreads within the large-scale gains category exhibiting cytological features associated with endoreduplication (Fig. S2). Collectively, these data demonstrate that reduced *EMI1* expression induces CIN in male HCT116 cells and includes both gains and losses in chromosome numbers.

### Reduced *EMI1* expression promotes CIN in a female CRC cell line

Previous CIN studies in colonic contexts have only employed male CRC cell lines, and therefore do not provide insight into potential sex differences. To address this limitation, we identified SW48 as a potential line for subsequent investigation. SW48 are derived from an 82-year-old white female with stage III adenocarcinoma that we first confirmed have a near-diploid (47) modal chromosome number; 47, XX, +7, dup[10q]t(22;14). We subsequently determined that SW48 are karyotypically stable over 3 months of continual passaging as assessed by spectral karyotyping (SKY) (Fig. S1; Table S1). We also performed western blot analysis to confirm SW48 cells express EMI1 and compared its abundance relative to that of all cell lines employed in this study (Fig. S3). As characterising the molecular determinants of CIN mandates the use of karyotypically stable (CIN-) cell lines, SW48 were identified as an ideal model in which to study the impact reduced *EMI1* expression has on CIN.

**Fig. 3:**
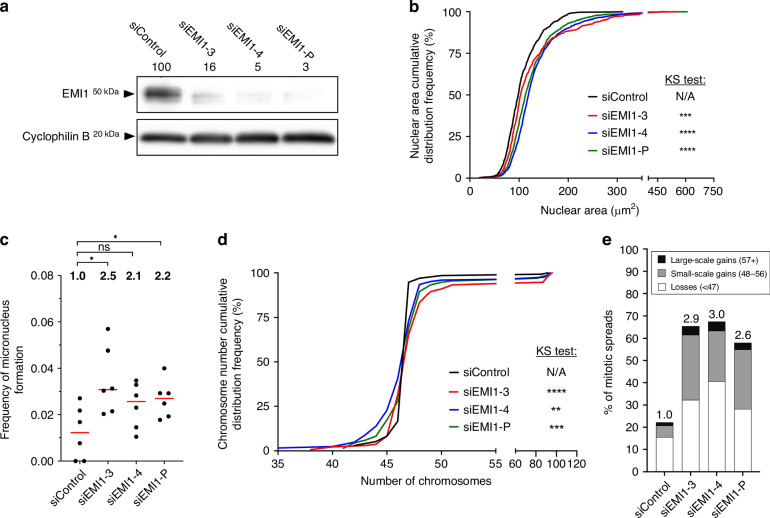
Diminished *EMI1* expression induces increases in CIN phenotypes in SW48 cells. **a** Semi-quantitative western blot presenting the silencing efficiency of si*EMI1*-3, -4, and -P in SW48. EMI1 abundance is normalised to the loading control and presented relative to siControl. **b** Cumulative distribution frequency graph reveals significant increases in nuclear areas following *EMI1* silencing (two-sample KS test; N/A, not applicable; ***p-value < 0.001, ****p-value < 0.0001). **c** Dot plot reveals increases in micronucleus formation following *EMI1* silencing. Median values are indicated by red bars, while fold increase relative to siControl are presented above each column (MW test; ns not significant *p*-value > 0.05; *p-value < 0.05; *n* = 3; 6 wells analysed/condition). **d** Chromosome number cumulative distribution graph identifies significant differences following *EMI1* silencing relative to siControl (two-sample KS test; N/A, not applicable, **p-value < 0.01, ***p-value < 0.001; ****p-value < 0.0001). **e** Bar graph presenting the frequencies of aberrant chromosome spreads following EMI1 silencing with the fold increase relative to siControl indicated above each bar (*n* = 3, ≥ 100 spreads analysed/condition).

As above, semi-quantitative western blots were performed using siEMI1-3, siEMI1-4, and siEMI1-P that reduced EMI1 abundance to ~3–16% of siControl cells (Fig. 3a). Subsequent QuantIM analyses identified significant increases in nuclear area distributions (Fig. 3b; Table S7) and significant (siEMI1-P, 2.2-fold increase and siEMI1-3, 2.5-fold) or tending (siEMI1-4, 2.1-fold) increases in micronucleus formation relative to siControl (Fig. 3c, Table S8). Finally, chromosome enumeration revealed significant differences in chromosome number distributions (Fig. 3d, Table S9) along with a 2.6- to 3.0-fold increase in aberrant chromosome spreads (Fig. 3e). Like HCT116, cytological features consistent with endoreduplication were observed in spreads harbouring large-scale gains; however, they were ~2-fold more common in SW48 cells (~60%) than HCT116 (~33%) (Fig. S2). Collectively, these findings support those of the preceding section and show that reduced *EMI1* expression induces CIN in SW48 cells, thus, identifying *EMI1* as a novel CIN gene in a female CRC context.

### Reduced *EMI1* expression induces CIN in non-malignant, non-transformed human colonic epithelial cells

**Fig. 4:**
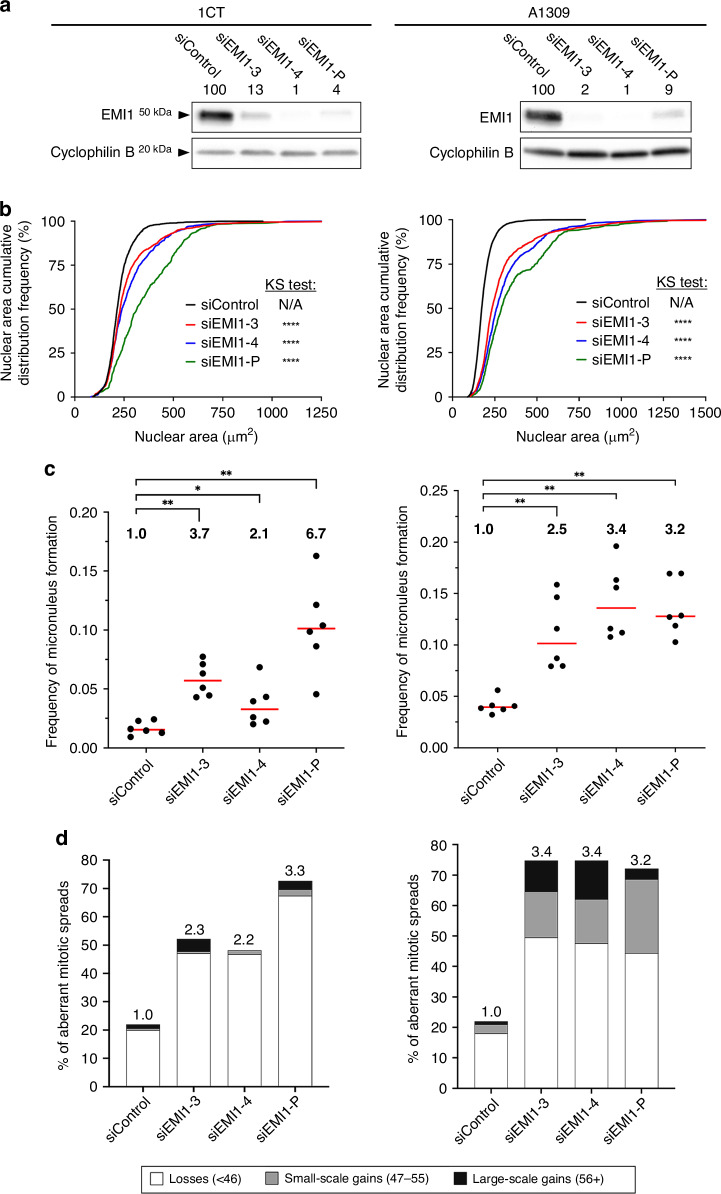
*EMI1* silencing underlies increases in CIN phenotypes in non-malignant/non-transformed colonic epithelial cells. **a** Semi-quantitative western blots presenting the silencing efficiency of si*EMI1*-3, -4 and -P in 1CT (left) and A1309 (right) cells. EMI1 abundance is normalised to the loading control (Cyclophilin B) and presented relative to siControl (100%). **b** Cumulative distribution frequency graphs reveal significant increases in nuclear area distributions (two-sample KS test) following *EMI1* silencing (two-sample KS test; N/A, not applicable; ****p-value < 0.0001; *n* = 3; 6 wells analysed/condition). **c** Dot plots reveal significant increases (MW test) in micronucleus formation relative to siControl (*p-value < 0.05, **p-value < 0.01; *n* = 3; 6 wells analysed/condition). Red bars identify median values (*n* = 3, 6 wells analysed/condition), while fold increase relative to siControl are presented at the top of each graph. **d** Bar graphs presenting the frequencies of aberrant chromosome spreads following silencing (*n* = 3; ≥ 100 spreads analysed/condition) with the fold increase indicated above each bar.

To determine whether reduced *EMI1* expression may contribute to early disease development, we silenced *EMI1* in non-malignant, non-transformed cellular contexts (i.e., models of early disease development) and assessed CIN as above. 1CT and A1309 cells were purposefully selected as they are karyotypically stable (modal number = 46), clinically relevant colonic epithelial cell lines that have also been employed in similar CIN-based studies [23, 36]. As above, *EMI1* silencing reduced endogenous protein levels to ~1–13% of siControl (Fig. 4a) and corresponded with increases in CIN phenotypes. More specifically, nuclear area distributions were significantly increased (Fig. 4b; Table S10) in both lines and were accompanied by significant 2.1- to 6.7-fold increases in micronucleus formation (Fig. 4c; Table S11). Mitotic chromosome spreads also revealed significant changes in chromosome number distributions (Fig. S4, Table S12) and a 2.2- to 3.4-fold increase in the frequencies of aberrant chromosome spreads (Fig. 4d). In general, the aberrant spreads included ~50% losses, ~1% small-scale gains, and <5% large-scale gains for 1CT and ~44% losses, ~18% small-scale gains, and ~3–16% large-scale gains for A1309. In agreement with the HCT116 and SW48 findings, evidence of endoreduplication was observed in ~40% (1CT) and ~16% (A1309) of aberrant spreads harbouring large-scale gains (Fig. S2). Collectively, these data show that *EMI1* silencing induces CIN phenotypes in 1CT and A1309 cells and identify *EMI1* as a novel CIN gene in non-malignant, non-transformed colonic epithelial cell contexts and are consistent with reduced expression contributing to early disease development.

### Generation and initial characterisation of clinically relevant *EMI1*^*+/*^^−^ models

As heterozygous loss of *EMI1* occurs in ~12% of all CRC patients and the above data support the possibility that reduced expression may be a pathogenic event, we next sought to assess the long-term impact *EMI1* loss has on CIN in clinically relevant models. *EMI1* appears to be an essential gene as homozygous losses of *EMI1* are extremely rare (~0.2%), and the Cancer Dependency Map (DepMap; https://depmap.org/portal/) [52] lists *EMI1* as a “common essential” gene. Accordingly, we generated clinically relevant, heterozygous knockout models to determine the long-term impact *EMI1* loss and reduced expression have on CIN and cellular transformation. A1309 were purposefully chosen as they are non-malignant, non-transformed cell line that have been engineered to contain additional predisposing genetic alterations (see Methods) typically occurring early in CRC development that may synergise with *EMI1* loss.

Using CRISPR/Cas9 approaches, we generated two heterozygous *EMI1* knockout clones, termed *EMI1*^+/^^−^1 and *EMI1*^+/^^−^2, that were validated through semi-quantitative western blots and DNA sequencing (Fig. S5). Briefly, EMI1 abundance was reduced to 37% (*EMI1*^+/^^−^1) and 40% (*EMI1*^+/^^−^2) of the non-targeting sgRNA control (NT-Control) clone (Fig. S5A), with *EMI1*^+/^^−^2 also exhibiting a slightly faster migrating band. DNA sequencing revealed that *EMI1*^+/^^−^1 harbours a 2 base pair (frameshift) deletion in one allele, while also retaining a single wild-type copy (Fig. S5B), whereas *EMI1*^+/^^−^2 is a compound heterozygote with a similar 2 base pair deletion in one allele and a 27 base pair (in-frame) deletion in the second allele (Fig. S5B). Subsequent in silico analyses (Fig. S5C) revealed that the 2 base pair deletion induces a premature stop codon that is predicted to induce nonsense-mediated mRNA decay and prevent protein production, while the 27 base pair deletion corresponds with a nine amino acid (~1 kDa) deletion and likely accounts for the faster migrating band, and presumably a partially functional protein as complete loss of function is expected to be lethal.

### Heterozygous loss of *EMI1* induces ongoing and dynamic CIN phenotypes in non-malignant, human colonic epithelial cells

**Fig. 5:**
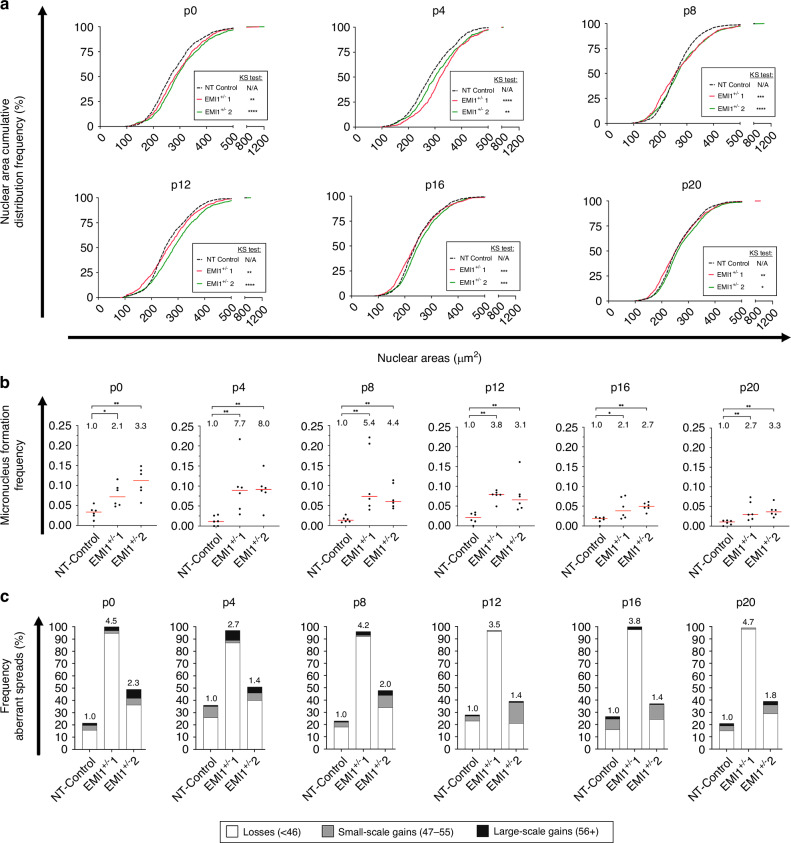
A1309 *EMI1*^+/^^−^ clones exhibit dynamic changes in CIN phenotypes over 20 passages. **a** Cumulative distribution frequency graphs reveal dynamic and significant changes in nuclear areas in both A1309 *EMI1*^+/^^−^ clones (*EMI1*^+/^^−^1 and *EMI1*^+/^^−^2) relative to NT-Control (two-sample KS test; N/A, not applicable, *p-value < 0.05; **p-value < 0.01; ***p-value < 0.001; ****p-value < 0.0001). The passage number (p) is indicated at the top of each graph (*n* = 1; 6 wells analysed/condition/time point). **b** Dot plots reveal significant increases (MW test) in micronucleus formation in *EMI1*^+/^^−^ clones relative to NT-Control. Median values are indicated by red bars, while the fold increase relative to NT-Control are presented at the top of each column (*p-value < 0.05; **p-value < 0.01; *n* = 1; 6 wells analysed/condition). **c** Bar graphs presenting the frequencies of aberrant chromosome spreads, including chromosome losses (< 46 chromosomes; white), small-scale gains (47–55; grey) and large-scale gains (≥ 56; black) relative to NT-Control (modal number = 46). Fold increases in the total frequencies of aberrant chromosome spreads are indicated above each bar (*n* = 1, ≥ 100 spreads/condition).

To determine the long-term impact heterozygous loss of *EMI1* has on CIN, both *EMI1*^+/^^−^ and NT-Control clones were continually passaged for 10 weeks, with serial aliquots assessed by QuantIM every four passages (p; approximately every 2 weeks). Consistent with CIN and ongoing genetic and cell-to-cell heterogeneity, both *EMI1*^+/^^−^ clones exhibited dynamic phenotypes from early (p0) to late (p20) passages (Fig. 5). More specifically, *EMI1*^+/^^−^1 exhibited significant increases in nuclear area distributions relative to NT-Control at p0 and p4 that later decreased from p8 until p20 but remained statistically significant (Fig. 5a, Table S13). On the other hand, *EMI1*^+/^^−^2 exhibited considerably larger nuclear areas at p0 that decreased slightly in p4 and p8 yet remained larger than those of the NT-Control. At p12, an increase in nuclear area distributions occurred, which decreased towards later passages (p16 and p20). With respect to micronucleus formation, both *EMI1*^+/^^−^ clones presented striking increases from p0 to p4; ~2.1-fold to ~7.7-fold, and ~3.3-fold to ~8.0-fold, respectively (Fig. 5b, Table S14) that tended to decrease at p8 through p16 (~5.4- and ~4.4-fold to ~2.1- and ~2.7-fold) and increased slightly at p20 (~2.7- and ~3.3-fold). While some fluctuations in nuclear area distributions occurred between passages for the NT-Control, these were not deemed statistically significant by ANOVA and Tukey multi-comparison post-tests (Tables S15, S16). Collectively, both *EMI1*^+/^^−^ clones exhibit ongoing and dynamic changes in nuclear area distributions and micronucleus formation, which is in agreement with heterozygous loss and reduced *EMI1* expression inducing CIN.

To determine whether heterozygous loss of *EMI1* corresponds with changes in chromosome numbers over time, a minimum of 100 mitotic chromosome spreads were manually enumerated at each passage from all clones. In general, both *EMI1*^+/^^−^ clones exhibited dynamic changes in chromosome complements that included both losses and gains (Fig. 5c; Fig. S6; Table S17). Remarkably, nearly all chromosome spreads from *EMI1*^+/^^−^1 at each passage harboured aberrant chromosome numbers, with ~90% of them being chromosome losses. In contrast, chromosome alterations in *EMI1*^+/^^−^2 were less pronounced and more dynamic, as the frequency of total aberrant spreads typically ranged between ~40% and 50% at each passage (Fig. 5c). Collectively, the ongoing and dynamic changes in nuclear area distributions, increased frequencies of micronucleus formation and the ongoing gains and/or losses of chromosome complement reveal that heterozygous loss and reduced *EMI1* expression induces CIN and is consistent with it being a contributing factor in early disease development.

### *EMI1* loss corresponds with increases in DNA DSBs and cellular transformation

**Fig. 6:**
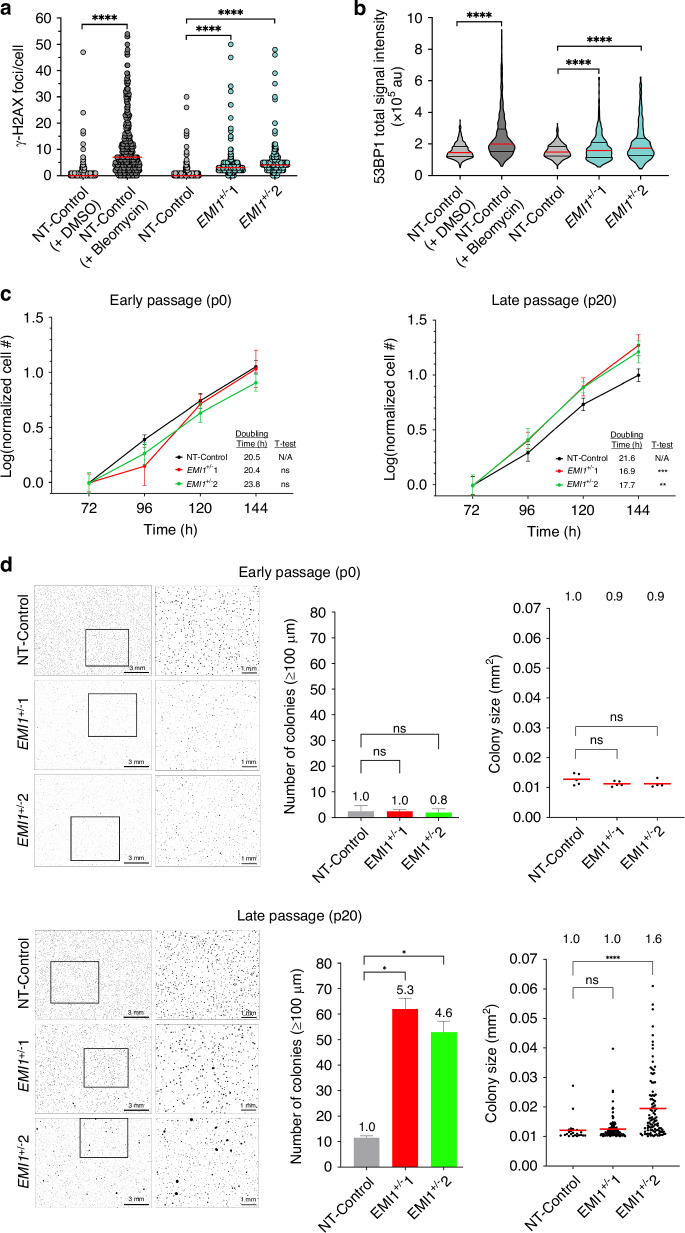
*EMI1* loss corresponds with increases in DNA DSBs and cellular transformation. **a** Scatter plot reveals significant increases in the number of γ-H2AX foci/cells within bleomycin-treated cells and within the *EMI1*^+/^^−^ clones relative to DMSO-treated and NT-Control clones, respectively. Red lines identify median values (one-sided MW tests; ****p-value < 0.0001; *n* = 1; > 200 nuclei/condition). **b** Violin plot identifies significant increases in 53BP1 total signal intensities in both bleomycin-treated cells and the *EMI1*^+/^^−^ clones relative to DMSO-treated and NT-Control clones, respectively. Violin plots present the overall and interquartile ranges (one-sided Student’s *t*-tests; ****p-value < 0.0001; *n* = 1; > 200 nuclei/condition). **c** Growth curves for early (p0; left) and late (p20; right) passage *EMI1*^*+/*^^−^ clones from 72–144 h post-seeding. Cell numbers are normalised to the mean cell number on day 3 (72 h), and data points show the mean cell number ± standard deviation (Multiple *t*-test; ns not significant; **p-value < 0.01; ***p-value < 0.001; *n* = 1; 6 wells analysed/condition/time point). **d** Low-resolution image (left) of colony formation with magnified regions (right) identified by the bounding boxes. Bar graphs (middle) presenting the mean (± standard deviation) number of colonies (operationally defined as ≥ 100μm in diameter and an area > 0.01 mm^2^) and dot plots (right) showing colony sizes at early (p0; top) and late (p20; bottom) passages. Fold increases in mean colony numbers and sizes are presented above each column, while red bars identify means (Welch’s *t*-test; ns not significant *p*-value > 0.05; *p-value < 0.05, ****p < 0.0001; *n* = 1, 2 wells analysed/condition/time point).

Having established that both *EMI1*^+/^^−^ clones exhibit CIN, we next sought to gain mechanistic insight into the underlying defects contributing to CIN and genome instability. Given that SCF^EMI1^ normally targets RAD51 for proteolytic degradation [27] and RAD51 regulation through ubiquitination and/or degradation ensures both its association and timely removal from DSBs [53], we employed QuantIM to assess the impact reduced *EMI1* expression has on DNA DSB repair. Using two surrogate markers of DSBs, namely γ-H2AX and 53BP1, we first confirmed our ability to detect significant changes in the number of γ-H2AX foci and 53BP1 total signal intensities (Fig. S7; Tables S18, S19) following bleomycin (radiomimetic drug that induces DNA DSBs) treatments in NT-Control cells relative to a DMSO-treated control (Fig. 6a, b). Using this approach, we next assessed asynchronously growing populations and noted significant increases in both γ-H2AX foci (Fig. 6a; Table S18) and 53BP1 total signal intensities (Fig. 6b; Table S19) within interphase cells for both *EMI1*^+/^^−^ clones relative to the NT-Control clone. Collectively, these data indicate that *EMI1* loss and reduced expression in the *EMI1*^+/^^−^ clones are associated with increases and/or the persistence of DSBs, which agrees with recent studies identifying roles for EMI1 in effective DSB repair [54, 55].

CIN is an enabling hallmark of cancer [56] and is proposed to be an early aetiological event in CRC as it can promote cellular transformation [10, 57]. Accordingly, we evaluated the impact heterozygous loss of *EMI1* has on key phenotypes of cellular transformation (i.e., changes in cellular proliferation and anchorage-independent growth) in both early (p0) and late (p20) passage populations. As shown in Fig. 6c, while both *EMI1*^+/−^ clones exhibit variable doubling times at p0, they were not statistically different from the NT-control (20.5 h), with *EMI1*^+/^^−^1 and *EMI1*^+/^^−^2 doubling times being 20.4 h and 23.8 h, respectively; however, both clones exhibited significantly faster doubling times (16.9 h and 17.7 h, respectively) relative to NT-Control (21.6 h) at p20 (Table S20). Similar differences in anchorage-independent growth were also noted at early (p0) versus late (p20) passages for the *EMI1*^+/^^−^ clones relative to NT-Control (Fig. 6d). For example, while no statistical differences in the number of colonies occurred at p0, significant increases were apparent at p20. More specifically, *EMI11*^+/^^−^1 exhibited a 5.3-fold increase in colony numbers, while *EMI1*^+/^^−^2 exhibited a 4.6-fold increase along with a significant 1.6-fold increase in mean colony size (0.019 mm^2^) relative to NT-Control (0.012 mm^2^; Tables S21, S22). No statistical difference in colony sizes was noted for *EMI1*^+/^^−^1 (0.011 mm^2^). Collectively, these data demonstrated that both *EMI1*^+/^^−^ clones gain cellular transformation phenotypes over time that are consistent with a contributing role in early disease development.

## Discussion

CIN is an aberrant phenotype suspected to contribute to early disease development, cancer progression and the acquisition of drug resistance and is frequently associated with poor patient outcomes [5–7, 10–14]. Despite this information, little is known about the molecular determinants giving rise to CIN, especially in CRC, where it occurs in ~85% of all cases [3]. In this study, we determined the impact reduced *EMI1* expression has on CIN and early CRC development. Using publicly available clinical datasets, we determined that heterozygous loss occurs in ~12% of CRC cases, is associated with reduced expression and coincides with increases in genome instability and worse patient outcomes. To functionally determine the impact reduced *EMI1* expression has in CIN, transient siRNA-based silencing was employed that revealed significant increases in three CIN phenotypes (nuclear areas; micronucleus formation; aberrant chromosome numbers) in four colonic epithelial cell lines—two malignant/transformed cell contexts (male HCT116 and female SW48) and two non-malignant/non-transformed cell contexts (1CT and A1309). As CIN drives ongoing genetic and cell-to-cell heterogeneity, clinically relevant heterozygous knockout clones were generated, and CIN was assessed in serially passaged cells over a 10-week period. In support of a CIN phenotype, both *EMI1*^+/^^−^ clones exhibited ongoing and dynamic changes in CIN phenotypes over time, including significant changes in nuclear areas, micronucleus formation and chromosome numbers. Furthermore, both clones exhibited higher basal levels of DNA DSBs as evidenced by increases in γ-H2AX foci and 53BP1 signals relative to NT-Controls that corresponded with cellular transformation phenotypes (i.e., enhanced proliferation and anchorage-independent growth) over time. Collectively, this work determined that reduced *EMI1* expression induces CIN, increases basal levels of DNA DSBs and promotes cellular transformation, which collectively supports the possibility that heterozygous loss may be a pathogenic event contributing to CRC development.

CIN is characterised by an increased rate of gains and/or losses of whole chromosomes or chromosome fragments [3]. As such, it was expected that reduced *EMI1* expression would induce both gains and losses of chromosome complements that would be identified as either large or small nuclear areas, respectively, and confirmed by mitotic chromosome spreads. However, while *EMI1* silencing consistently induced increases in nuclear areas across all cell lines, chromosome enumeration showed higher frequencies of losses than gains. This perceived discrepancy may be explained, at least in part, by the differences between the assays and the experimental conditions in which they are performed. Briefly, nuclear area analyses are conducted on asynchronous populations in which most (>90%) cells are in interphase (G1, S-phase, G2), and mitotic cells are excluded from the analyses as they lack a nuclear envelope. Conversely, mitotic chromosome spreads only evaluate cells capable of entering and becoming enriched within mitosis following a brief colcemid treatment. Thus, each assay is inherently different as they quantitatively assess distinct phenotypes in disparate cellular populations, underscoring the need to employ multiple, complementary CIN assays. Moreover, the nuclear area analyses revealed striking increases across all cell lines following *EMI1* silencing, which may be partially explained by increases in endoreduplication, which have been observed by others [58]. Accordingly, as cells undergoing endoreduplication aberrantly re-replicate their DNA without entering mitosis, these populations will be more readily captured in the nuclear area analyses, while only a subset will eventually progress into mitosis, where they will be captured in chromosome spreads.

Overall, the role reduced *EMI1* expression has in endoreduplication may be associated with the EMI1-dependent inhibition of APC/C^CDH1^ (APC/C bound to CDH1 [E-cadherin]) [58], as it prevents the destabilization and premature degradation of geminin and Cyclin A (inhibitors of replication origin licensing) [58] to ensure proper mitotic entry. However, Cyclin E1 (*CCNE1*) is not degraded in an APC/C-dependent manner, which results in its increased abundance in the absence of EMI1 [59]. *CCNE1* is an established oncogene whose genomic amplification, overexpression and aberrant accumulation are associated with cell cycle misregulation, genome instability and tumour formation in mice [11, 60, 61]. Previous research concluded that reduced expression of *SKP2*, an F-box protein, led to aberrant Cyclin E1 accumulation [23], which in turn phenotypically mimics *CCNE1* overexpression and promotes endoreduplication. A crucial link between *EMI1* expression and SKP2 stabilisation may explain the increase in endoreduplication events as SKP2 is stabilised in an EMI1-dependent manner, where EMI1 competes for binding of APC/C^CDH1^, thereby inhibiting APC/C^CDH1^-mediated ubiquitination and subsequent degradation of SKP2 [62]. Indeed, *EMI1* silencing has confirmed this relationship as reduced EMI1 abundance correlated with reduced SKP2 protein abundance [62]. So, while EMI1 has traditionally been described as an oncogene—it is frequently overexpressed in many cancer types where it is associated with disease development, progression, therapeutic resistance and poor patient outcomes [31, 63, 64]—our data are consistent with *EMI1* also possessing tumour suppressor-like properties, as its reduced expression induces CIN and cellular transformation. Although this remains to be formally tested, a possible tumour suppressive role may arise through its relationship with SKP2, where EMI1 prevents the uncontrolled degradation of SKP2 by APC/C^CDH1^, thereby allowing SCF^SKP2^ to regulate Cyclin E1 abundance preventing its aberrant accumulation. Collectively, these studies highlight a potential mechanism by which *EMI1* loss may induce aberrant Cyclin E1 accumulation and contribute to CIN, promote cellular transformation, and underlie disease development.

Generating novel *EMI1* knockout models was essential to assess the long-term impact reduced *EMI1* expression has on CIN and early disease development, and although *EMI1*^*+/*^^−^1 and *EMI1*^*+/*^^−^2 exhibit similar EMI1 protein abundance (~40%), QuantIM analyses revealed both clones exhibited distinct, heterogeneous CIN phenotypes. While such differences may appear counterintuitive, these diverse outcomes are expected, given that CIN will induce ongoing and random karyotypic evolution in distinct cellular populations. In this regard, both *EMI1*^*+/*^^−^ clones exhibited significant changes in nuclear area distributions and increases in micronucleus formation frequencies at all time points relative to NT-Control; however, the changes varied between clones. Remarkably, the most dramatic differences observed between *EMI1*^*+/*^^−^ clones occurred in the frequencies of aberrant mitotic chromosome spreads, as nearly 100% of spreads from *EMI1*^*+/*^^−^1 were aberrant, whereas only 40–50% of those from *EMI1*^*+/*^^−^2 were aberrant. Although speculative, such striking differences in chromosome numbers may have originated from an early clonal expansion event underlying karyotypic variation between the clones despite being generated in the same parental cell line (A1309). Additionally, the long-term assays comparing early (p0) and late (p20) time points revealed that the *EMI1*^*+/*^^−^ clones acquired cellular transformation phenotypes over time. For example, the proliferation assays revealed that the *EMI1*^*+/*^^−^ clones exhibited faster doubling times at p20 relative to NT-Control. These findings contrast with those of Zhang et al. [65], who showed that loss of *EMI1* in gastric cancer cells corresponded with slower proliferation rates and reduced penetration capabilities. However, it should be noted that their results were obtained using homozygous (*EMI1*^−^^*/*^^−^*)* knockout models, which contrasts with DepMap data indicating *EMI1* is a common essential gene [52] but does support the possibility of context-specific essentiality. In the current study, the faster doubling times noted for both *EMI1*^*+/*^^−^ clones at p20 indicate that heterozygous loss promotes increased cellular proliferation over time in a colonic epithelial cell context. Additionally, the anchorage-independent growth assays revealed significant increases in mean colony numbers and sizes in *EMI1*^*+/*^^−^ clones from p0 to p20, indicating that the clones also acquired 3D growth capabilities with time. Although this study only captures a short time frame of a disease that typically requires 10–15 years to develop [66, 67], it highlights the impact heterozygous loss of *EMI1* has in a relatively short period of time (~10 weeks).

In summary, our data identified *EMI1* as a novel CIN gene, as reduced expression induces increases in CIN phenotypes and markers of DNA DSBs that promote cellular transformation, which is consistent with a role in early CRC development. Further studies aimed at determining the specific mechanisms underlying CIN, such as the SKP2 relationship detailed above and the tumourigenic potential following *EMI1* loss, are now essential to elucidate the molecular mechanism(s) giving rise to CIN. Finally, while our study is acutely focused on CRC, our findings may have broad spectrum implications as *EMI1* copy number losses occur in many cancer types, including breast, ovarian, prostate, and lung, although this remains to be formally tested.

## Supplementary information

Supplementary Information

## Data Availability

Patient-related data (Fig. 1) are based upon data generated by the TCGA Research Network and are available at https://cancer.gov/tcga or through https://www.cbioportal.org. All descriptive statistics and statistical analyses presented in Figs. 2–6,  S1, S4, S6 and S7 are provided in Supplementary Materials, Tables S1 and S4–S22.
