# Supplementary material for: Loss of EMI1 compromises chromosome stability and is associated with cellular transformation in colonic epithelial cell contexts
Source: Br J Cancer. 2024 Oct 2;131(9):1516–28. doi: 10.1038/s41416-024-02855-9 (PMC11519589; doi:10.1038/s41416-024-02855-9)
Supplement: Supplementary file 1 — Supplementary Information [file 41416_2024_2855_MOESM1_ESM.pdf]

SUPPLEMENTARY MATERIALS

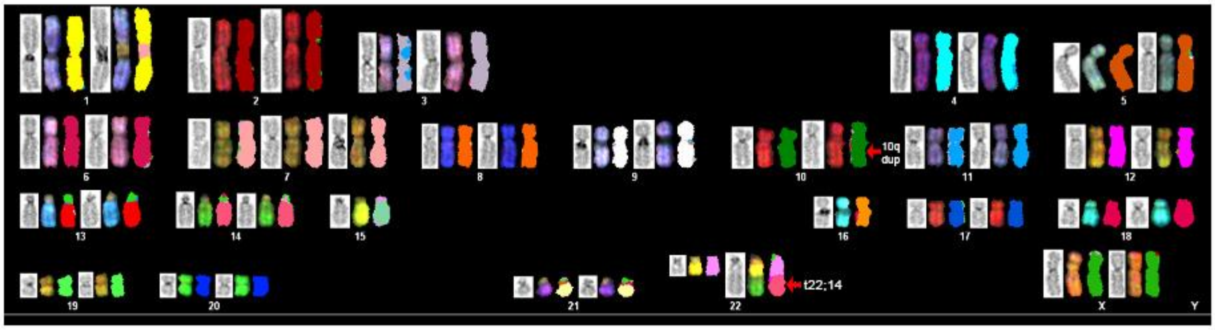

Figure S1. Spectral karyotyping of the SW48 CRC cell line.
Representative example of the modal spectral karyotype for SW48; 47, xx, +7, dup(10q)t(22;14). The red arrows identify the regions of recurrent duplication (dup[10q]) and translocation (t[22;14]) events.

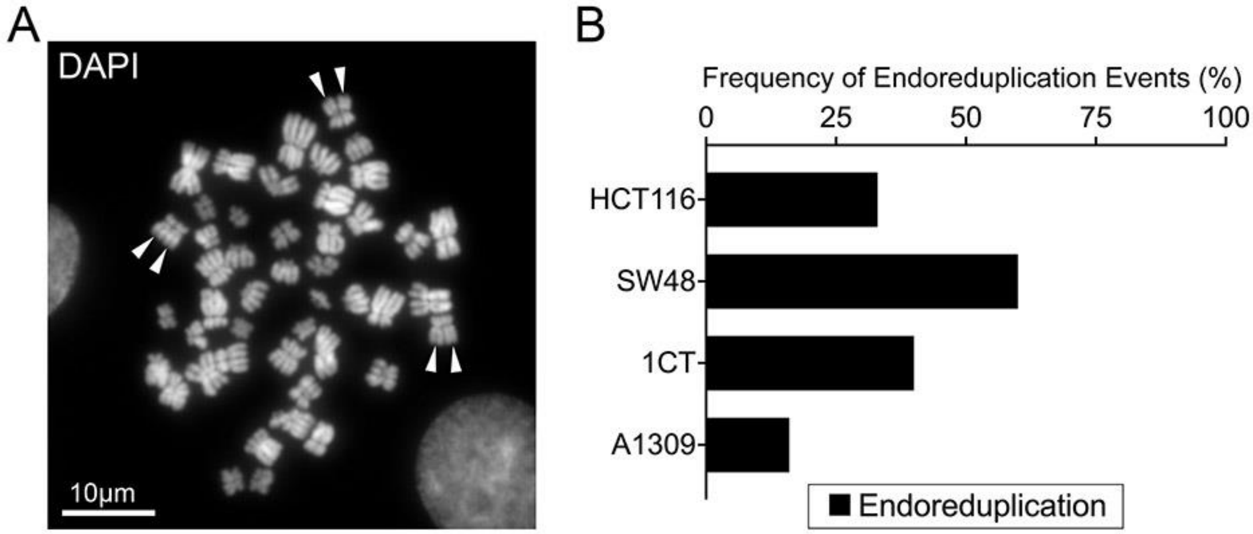

Figure S2. Reduced EMI1 abundance corresponds with increases in endoreduplication.
(A) Representative high-resolution image of a DAPI-counterstained mitotic chromosome spread depicting an endoreduplication event following *EMI1* silencing in HCT116 cells. The pairing of chromosomes (pairs of paired sister chromatids) is indicative of endoreduplication. For illustrative purposes, 3 pairs of paired sister chromatids are highlighted with arrowheads. (B) Bar graph presenting the frequencies of total endoreduplication events within the large scale gains category following *EMI1* silencing within the HCT116, SW48, 1CT and A1309 cell lines.

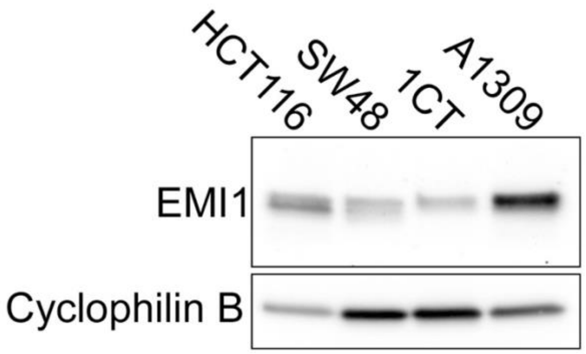

Figure S3. Western blot comparing basal EMI1 levels in four asynchronously growing colonic epithelial cell lines.

Protein extracts from each of the four cell lines (HCT116; SW48; 1CT; A1309) were harvested from asynchronously growing cultures and subjected to western blot analysis. The membrane was blotted for EMI1, while Cyclophilin B was used as the loading control.

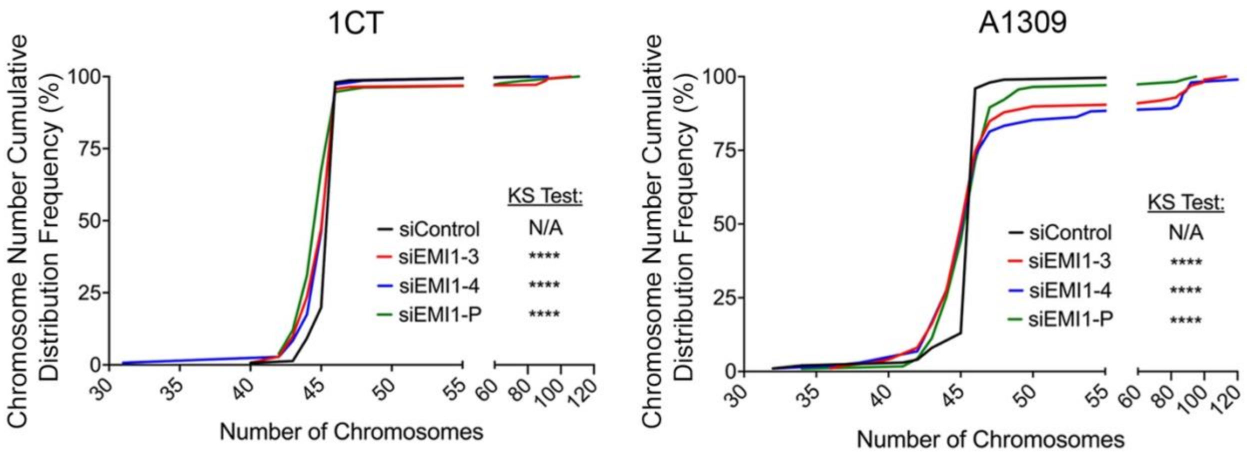

Figure S4. *EMI1* silencing induces underlies increases in chromosome number distributions in 1CT and A1309 cells.

Cumulative distribution frequency graphs reveal significant changes in chromosome number distributions in si*EMI1* conditions compared to siControl in 1CT and A1309 cells (Two-sample KS test; N/A, not applicable; **** p-value < 0.0001). Descriptive statistics presented in Table S12.

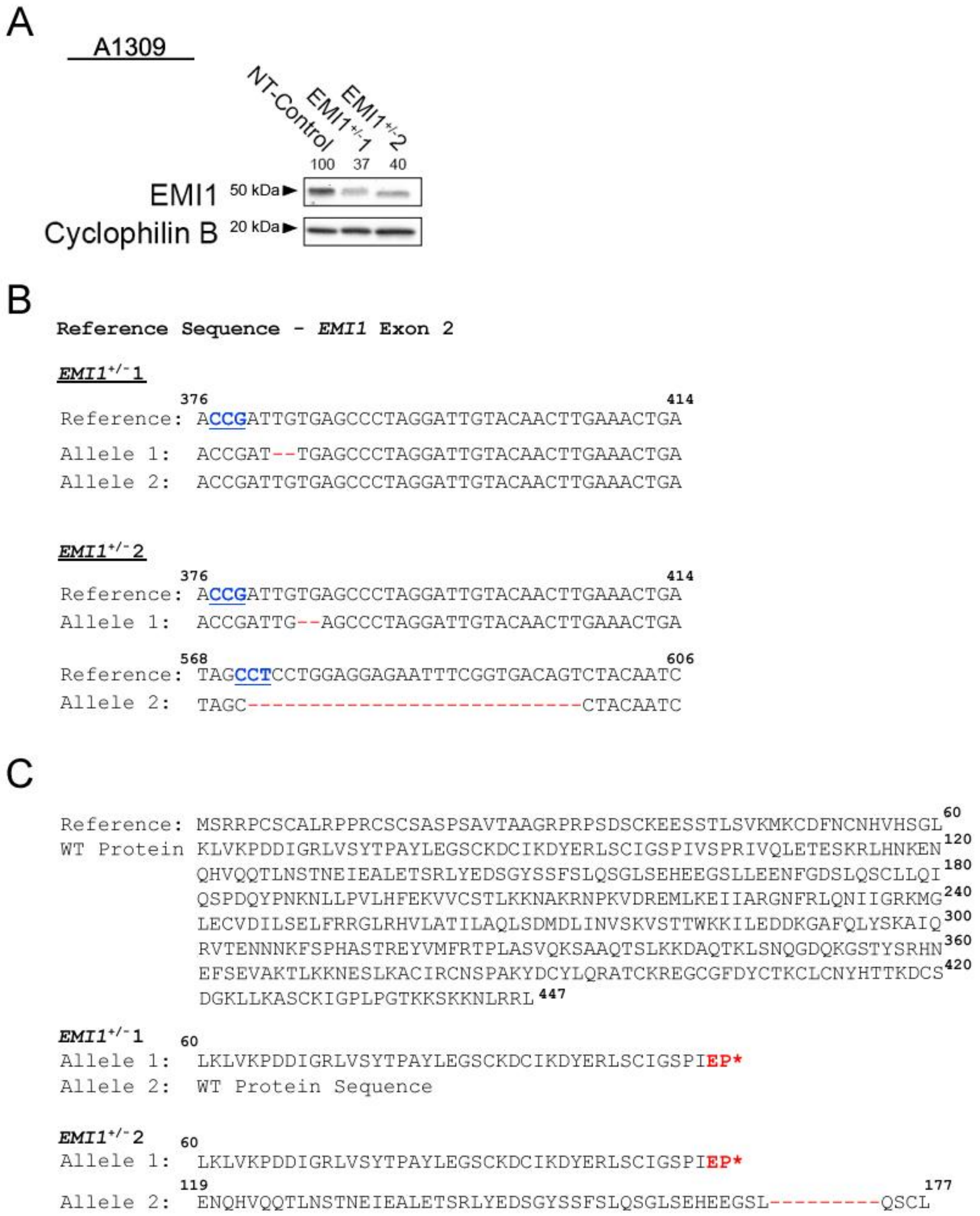

Figure S5. Characterization of the *EMI1* CRISPR edits within the A1309 cell line.
(A) Western blot presenting the abundance of EMI1 in *EMI1*$^{+/-}$1 (37%) and *EMI1*$^{+/-}$2 (40%) clones relative to NT-Control (100%); Cyclophilin B serves as a loading control. (B) DNA sequencing identified a 2 base pair deletion within *EMI1*$^{+/-}$1 and *EMI1*$^{+/-}$2, while *EMI1*$^{+/-}$2 also harbours an additional 27 base pair deletion resulting in a 9 amino acid deletion in EMI1. The protospacer adjacent motif (PAM) is identified in blue and underlined. (C) Presented are the putative EMI1 amino acid sequences encoded by the CRISPR edits relative to the reference sequence (NP_036309.1), with modified amino acids indicated in red, and the asterisks (*) identifying premature stop codons. Note that nonsense mediated mRNA decay is predicted to prevent production of the prematurely truncated forms of EMI1, given the position of the 2 base pair (frameshift) edits within the corresponding transcripts.

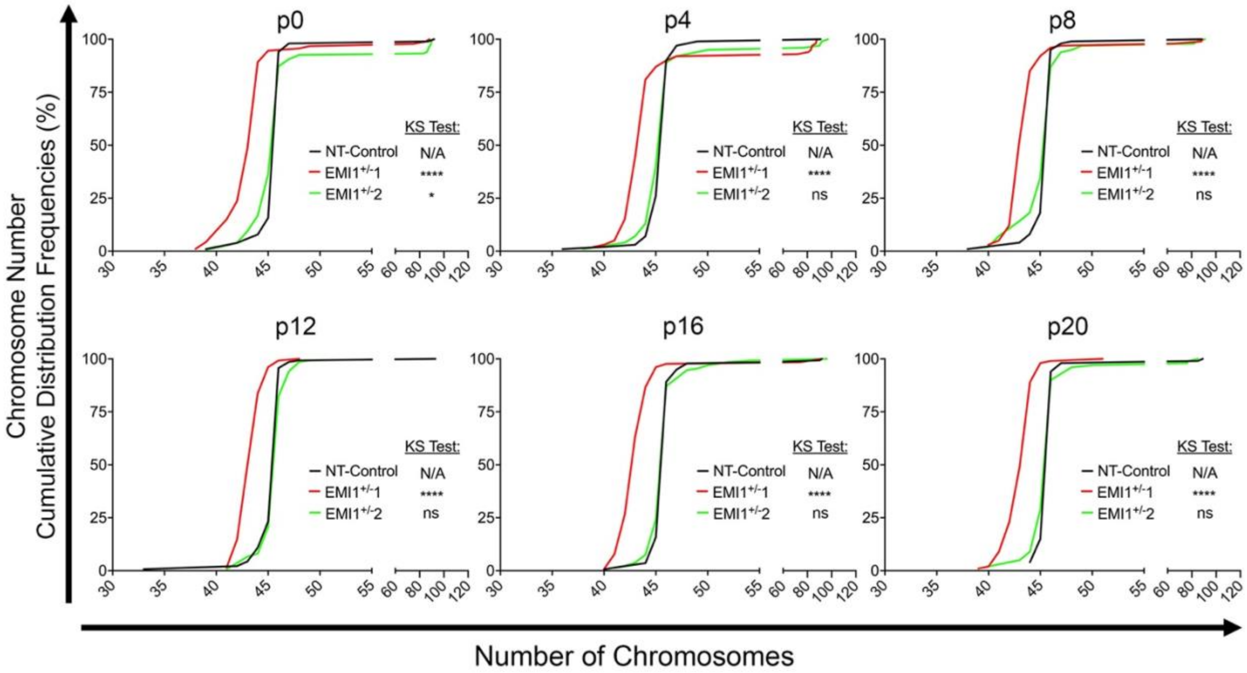

Figure S6. Heterozygous loss of *EMI1* induces changes in chromosome number distributions over time.

(A) Cumulative distribution frequency reveal *EMI1*$^{+/-}$ clones exhibit significant changes in chromosome number distributions relative to NT-control over time (Two-sample KS test; N/A, not applicable; ns, not significant p-value > 0.05; **** p-value < 0.0001). Passage number (p) is indicated above each graph. Descriptive statistics presented in Table S17.

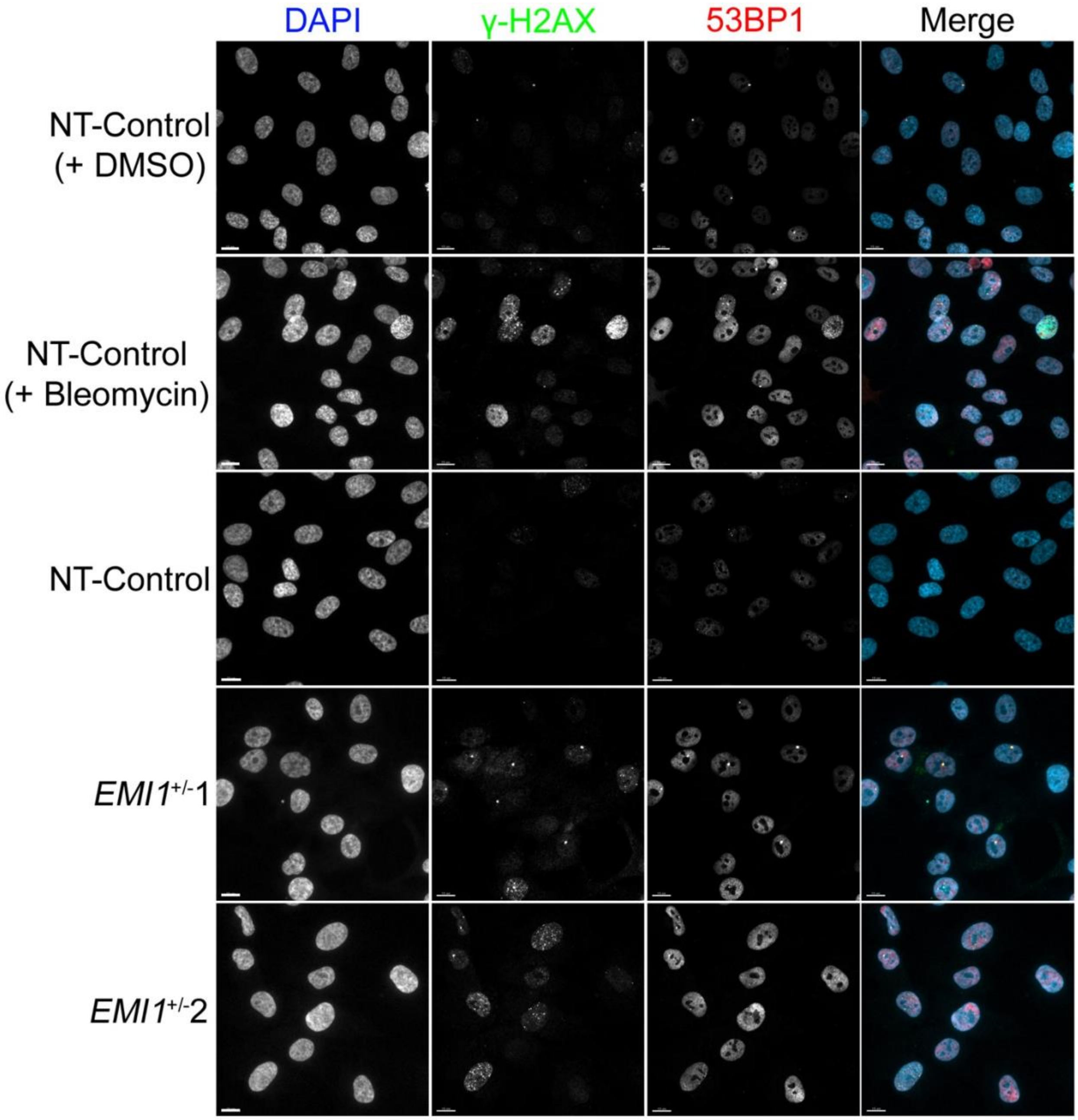

Figure S7. Asynchronously growing EMI1+/- clones harbour increased levels of DNA DSBs. Representative low-resolution images of γ-H2AX and 53BP1 labeling in NT-Control cells treated with and without bleomycin (0.1μg/ml; 2 h) and in NT-Control and the *EMI1*$^{+/-}$ clones. Exposure times for each channel were maintained constant throughout the acquisition phase. Scale bars represent 15μm.

Table S1. Enumeration of mitotic chromosome spreads reveal SW48 is a karyotypically stable cell line.

Time Point (Months)	n[A]	MCS with modal number (%)[B]
0	101	79.2%
1	101	68.3%
2	101	90.1%
3	100	87.0%

[A]Number of mitotic chromosome spreads analyzed
[B]Percentage of mitotic chromosome spreads that exhibit a modal number of 47 chromosomes

Table S2. Antibodies and dilutions employed for western blots.

Antibody	Source[A]	Cat. No.	Species	Dilution
EMI1	Abcam	ab215765	Mouse	1:1500
RAD51	Abcam	ab213	Mouse	1:5000
Cyclophilin B	Abcam	ab16045	Rabbit	1:150,000
Goat α Mouse HRP[B]	JIR	115-035-146	Goat	1:10,000
Goat α Rabbit HRP[B]	JIR	111-035-144	Goat	1:15,000

[A]JIR, Jackson ImmunoResearch
[B]HRP, horseradish peroxidase

Table S3. sgRNA sequences and *EMI1* target sites.

sgRNA	Sequence[A]	Target Site
sgNT	5'-CGCGAUAGCGCGAAUAUAUU-3'	None
sg*EMI1*-1	5'-ACCGAAATTCTCCTCCAGG-3'	*EMI1* Exon 2
sg*EMI1*-2	5'-CAATCCTAGGGCTCACAAT-3'	*EMI1* Exon 2

[A] Each sgRNA is composed of a variable 20 nucleotide sequence at its 3'end (shown above), which either does not target any sequences in the human genome (sgNT) or targets a unique complementary region within the *EMI1* coding sequence (sg*EMI1*-1 or sg*EMI1*-2). The following 82 nucleotide sequence at the 5' end of each sgRNA enables ribonucleoprotein complex formation with the Cas9 endonuclease: 5'-UUUUUUCGUGGCUGAGCCACGGUGAAAAAGUUCAAC UAUUGCCUGAUCGGAAUAAAAUUGAACGAUAAAGAUCGAGAUUUUG-3'.

Table S4. Two-sample KS tests reveal significant increases in cumulative nuclear area distribution frequencies following *EMI1* silencing in HCT116 cells.

Condition	n[A]	p-value[B]	Sig[C]
siControl	> 300	N/A	N/A
si*EMI1*-3	> 300	< 0.0001	****
si*EMI1*-4	> 300	< 0.0001	****
si*EMI1*-Pool	> 300	< 0.0001	****

[A]Number of nuclei analyzed
[B]p-values calculated from two-sample KS tests for the listed condition relative to siControl (non-targeting siRNA); N/A, not applicable
[C]Sig, Significance; ****, p-value < 0.0001

Table S5. MW tests identify significant increases in micronucleus formation frequencies following *EMI1* silencing in HCT116 cells.

Condition	n[A]	Mean Nuclear Count[B]	Mean MN Count[C]	Mean % MNF[D]	Median Fold Change in MNF[E]	p-value[F]	Sig[G]
siControl	6	667	20.0	3.1	N/A	N/A	N/A
si*EMI1*-3	6	159	38.2	24.9	7.7	0.0022	**
si*EMI1*-4	6	165	35.0	23.7	7.9	0.0022	**
si*EMI1*-Pool	6	156	33.2	23.5	7.3	0.0022	**

[A]n, number of wells analyzed
[B]Number of nuclei analyzed per well
[C]Mean number of micronuclei counted per well
[D]Mean percent MNF (calculated for each well as the MN count / nucleus count × 100)
[E]Median fold change in MNF relative to non-targeting control (siControl); N/A, not applicable
[F]p-values calculated from two-sample MW tests for the listed condition relative to non-targeting control at the corresponding time point
[G]Sig, Significance; **, p-value < 0.01

Table S6. Two-sample KS tests identify significant changes in chromosome number cumulative distribution frequencies following *EMI1* silencing in HCT116 cells.

Condition	n[A]	p-value[B]	Sig[C]
siControl	100	N/A	N/A
si*EMI1*-3	100	< 0.0001	****
si*EMI1*-4	100	0.0002	***
si*EMI1*-Pool	100	< 0.0001	****

[A]Number of mitotic chromosome spreads analyzed
[B]p-values calculated from two-sample KS tests for the listed condition relative to siControl (non-targeting siRNA); N/A, not applicable
[C] Sig, Significance; ***, p value < 0.001; ****, p-value < 0.0001

Table S7. Two-sample KS tests reveal significant increases in nuclear area cumulative frequency distributions following *EMI1* silencing in SW48 cells.

Condition	n[A]	p-value[B]	Sig[C]
siControl	> 300	N/A	N/A
si*EMI1*-3	> 300	0.0017	**
si*EMI1*-4	> 300	< 0.0001	****
si*EMI1*-Pool	> 300	< 0.0001	****

[A]Number of nuclei analyzed
[B]p-values calculated from two-sample KS tests for the listed condition relative to siControl (non-targeting siRNA); N/A, not applicable
[C] Sig, Significance; **, p-value < 0.01; ****, p-value < 0.0001

Table S8. MW tests identify significant increases in micronucleus formation frequencies following *EMI1* silencing in SW48 cells.

Condition	n[A]	Mean Nucleus Count[B]	Mean MN Count[C]	Mean % MNF[D]	Median Fold Change in MNF[E]	p-value[F]	Sig[G]
siControl	6	160	2.6	1.2	N/A	N/A	N/A
si*EMI1*-3	6	104	3.2	3.5	2.5	0.0260	*
si*EMI1*-4	6	244	5.6	2.4	2.1	0.0887	ns
si*EMI1*-Pool	6	138	3.2	2.7	2.2	0.0390	*

[A]Number of wells analyzed
[B]Number of nuclei analyzed per well
[C]Mean number of micronuclei counted per well
[D]Mean percent MNF (calculated for each well as the MN count / nucleus count × 100)
[E]Median fold change in MNF relative to non-targeting control (siControl); N/A, not applicable
[F]p-values calculated from two-sample MW tests for the listed condition relative to non-targeting control at the corresponding time point
[G]Sig, Significance; ns, not significant, p-value > 0.05; *, p-value < 0.05

Table S9. Two-sample KS tests identify significant changes in chromosome number cumulative distribution frequencies following *EMI1* silencing in SW48 cells.

Condition	n[A]	p-value[B]	Sig[C]
siControl	100	N/A	N/A
si*EMI1*-3	100	< 0.0001	****
si*EMI1*-4	100	0.0012	**
si*EMI1*-Pool	100	0.0003	***

[A]Number of mitotic chromosome spreads analyzed
[B]p-values calculated from two-sample KS tests for the listed condition relative to siControl (non-targeting siRNA); N/A, not applicable
[C]Sig, Significance; **, p-value < 0.01; ***, p value < 0.001; ****, p-value < 0.0001

Table S10. Two-sample KS tests identify significant increases in nuclear area cumulative distribution frequencies following *EMI1* silencing in 1CT and A1309 cells.

Cell Line	Condition	n[A]	p-value[B]	Sig[C]
1CT	siControl	> 300	N/A	N/A
	si*EMI1*-3	> 300	< 0.0001	****
	si*EMI1*-4	> 300	< 0.0001	****
	si*EMI1*-Pool	> 300	< 0.0001	****
A1309	siControl	> 300	N/A	N/A
	si*EMI1*-3	> 300	< 0.0001	****
	si*EMI1*-4	> 300	< 0.0001	****
	si*EMI1*-Pool	> 300	< 0.0001	****

[A]Number of nuclei analyzed

[B]p-values calculated from two-sample KS tests for the listed condition relative to siControl (non-targeting siRNA); N/A, not applicable

[C]Sig, Significance; ****, p-value < 0.0001

Table S11. MW tests identify significant increases in micronucleus formation frequencies following *EMI1* silencing in 1CT and A1309 cells.

Cell Line	Condition	n[A]	Mean Nucleus Count[B]	Mean MN Count[C]	Mean % MNF[D]	Median Fold Change in MNF[E]	p-value[F]	Sig[G]
1CT	siControl	6	309	5.0	1.7	N/A	N/A	N/A
	si*EMI1*-3	6	196	11.1	6.0	3.7	0.0022	**
	si*EMI1*-4	6	124	4.1	3.7	2.1	0.0260	*
	si*EMI1*-Pool	6	118	7.3	10.3	6.7	0.0022	**
A1309	siControl	6	880	7.2	4.1	N/A	N/A	N/A
	si*EMI1*-3	6	252	16.9	11.1	2.5	0.0022	**
	si*EMI1*-4	6	163	14.0	13.7	3.4	0.0022	**
	si*EMI1*-Pool	6	115	10.5	13.6	3.1	0.0022	**

[A]Number of wells analyzed

[B]Number of nuclei analyzed per well

[C]Mean number of micronuclei counted per well

[D]Mean percent MNF (calculated for each well as the MN count / nucleus count × 100)

[E]Median fold change in MNF relative to non-targeting control (siControl); N/A, not applicable

[F]p-values calculated from two-sample MW tests for the listed condition relative to non-targeting control at the corresponding time point

[G]Sig, Significance; *, p -value < 0.05; **, p-value < 0.01

Table S12. Two-sample KS tests identify significant changes in chromosome number cumulative distribution frequencies following *EMI1* silencing in 1CT and A1309 cells.

Cell Line	Condition	n[A]	p-value[B]	Sig[C]
1CT	siControl	100	N/A	N/A
	si*EMI1*-3	100	< 0.0001	****
	si*EMI1*-4	100	< 0.0001	****
	si*EMI1*-Pool	100	< 0.0001	****
A1309	siControl	100	N/A	N/A
	si*EMI1*-3	100	< 0.0001	****
	si*EMI1*-4	100	< 0.0001	****
	si*EMI1*-Pool	100	< 0.0001	****

[A]Number of mitotic chromosome spreads analyzed
[B]p-values calculated from two-sample KS tests for the listed condition relative to siControl (non-targeting siRNA); N/A, not applicable
[C]Sig, Significance; ****, p-value < 0.0001

Table S13. Two-sample KS tests reveal significant increases in nuclear area distributions in *EMI1*$^{+/-}$ clones over time.

Passage	Condition	p-value[A]	Sig[B]
p0	NT-Control	N/A	N/A
	EMI1$^{+/-}$1	0.0008	***
	EMI1$^{+/-}$2	< 0.0001	****
p4	NT-Control	N/A	N/A
	EMI1$^{+/-}$1	< 0.0001	****
	EMI1$^{+/-}$2	0.0043	**
p8	NT-Control	N/A	N/A
	EMI1$^{+/-}$1	0.0006	***
	EMI1$^{+/-}$2	< 0.0001	****
p12	NT-Control	N/A	N/A
	EMI1$^{+/-}$1	0.0060	**
	EMI1$^{+/-}$2	< 0.0001	****
p16	NT-Control	N/A	N/A
	EMI1$^{+/-}$1	0.0003	***
	EMI1$^{+/-}$2	0.0007	***
p20	NT-Control	N/A	N/A
	EMI1$^{+/-}$1	0.0001	***
	EMI1$^{+/-}$2	0.0015	**

[A]p-values calculated from two-sample KS tests for the listed condition relative to NT-control; N/A, not applicable
[B]Sig, Significance; **, p-value < 0.01; ***, p-value < 0.001; ****, p-value < 0.0001

Table S14. MW tests uncover significant increases in micronucleus formation frequencies in *EMI1*$^{+/-}$ clones over time.

Passage	Condition	n^A	p-value^B	Sig^C
p0	NT-Control	6	N/A	N/A
	EMI1$^{+/-}$1	6	0.0152	*
	EMI1$^{+/-}$2	6	0.0022	**
p4	NT-Control	6	N/A	N/A
	EMI1$^{+/-}$1	6	0.0087	**
	EMI1$^{+/-}$2	6	0.0087	**
p8	NT-Control	6	N/A	N/A
	EMI1$^{+/-}$1	6	0.0022	**
	EMI1$^{+/-}$2	6	0.0022	**
p12	NT-Control	6	N/A	N/A
	EMI1$^{+/-}$1	6	0.0022	**
	EMI1$^{+/-}$2	6	0.0022	**
p16	NT-Control	6	N/A	N/A
	EMI1$^{+/-}$1	6	0.0411	*
	EMI1$^{+/-}$2	6	0.0022	**
p20	NT-Control	6	N/A	N/A
	EMI1$^{+/-}$1	6	0.0022	**
	EMI1$^{+/-}$2	6	0.0022	**

^{A}Number of wells analyzed
^{B}p-values calculated from two-sample KS tests for the listed condition relative to NT-control; N/A, not applicable
^{C} Sig, Significance; *, p-value <0.05; **, p-value < 0.01

Table S15: Analysis of variance (ANOVA) of unweighted nuclear area means for A1309 cells at various passages.

Group^A	SS^B ($\times10^3$)	DF^C	MS^D	F Ratio^E	p-value	Reject H$_0$^{C}
BG	2.159	5	431.7	0.06895	0.9967	No
WG	46743	7465	6262			

^{A}defines whether the analysis is between groups (BG) or within the groups (WG)
^{B}Sum of squares (SS)
^{C}Degrees of freedom (DF)
^{D}Mean square (MS)
^{E}F ratio = MS_{BG}/MS_{WG}
^{C}The null hypothesis (H_0) is rejected if p-value <0.05

Table S16: Tukey multi-comparison post-test for differences in nuclear areas in A1309 cells at various passages.

	Sig[A]				
Passage	p4	p8	p12	p16	p20
p0	ns	ns	ns	ns	ns
p4		ns	ns	ns	ns
p8			ns	ns	ns
p12				ns	ns
p16					ns

[A]Sig, significance; ns, not significant, p-value > 0.05

Table S17. Two-sample KS tests identify significant changes in chromosome number cumulative distributions frequencies in *EMI1*$^{+/-}$ clones.

Passage	Condition	n[A]	p-value[B]	Sig[C]
p0	NT-Control	100	N/A	N/A
	EMI1$^{+/-}$1	100	<0.0001	****
	EMI1$^{+/-}$2	100	0.0120	*
p4	NT-Control	100	N/A	N/A
	EMI1$^{+/-}$1	100	<0.0001	****
	EMI1$^{+/-}$2	100	0.2809	ns
p8	NT-Control	100	N/A	N/A
	EMI1$^{+/-}$1	100	<0.0001	****
	EMI1$^{+/-}$2	100	0.1402	ns
p12	NT-Control	100	N/A	N/A
	EMI1$^{+/-}$1	100	<0.0001	****
	EMI1$^{+/-}$2	100	0.1772	ns
p16	NT-Control	100	N/A	N/A
	EMI1$^{+/-}$1	100	<0.0001	****
	EMI1$^{+/-}$2	100	0.7238	ns
p20	NT-Control	100	N/A	N/A
	EMI1$^{+/-}$1	100	<0.0001	****
	EMI1$^{+/-}$2	100	0.2809	ns

[A]Number of mitotic chromosome spreads analyzed

[B]p-values calculated from two-sample KS tests for the listed condition relative to NT-Control; N/A, not applicable

[C]Sig, Significance; ns, not significant, p-value > 0.05; *, p-value < 0.05; ****, p-value < 0.0001

Table S18: *EMI1*$^{+/-}$ clones exhibit significantly higher basal levels of γ-H2AX foci than NT-Control.

Condition	n[A]	Number of γ-H2AX Foci/Cell (Percentile)			Sig[B]
		25th	50th	75th	
NT-Control (DMSO)	404	0.000	0.000	1.000	N/A
NT-Control +Bleomycin	405	3.000	7.000	14.000	****
NT-Control	352	0.000	0.000	1.000	N/A
EMI1$^{+/-}$1	389	2.000	3.000	4.000	****
EMI1$^{+/-}$2	446	3.000	4.000	5.000	****

[A]n, number of interphase cells analyzed

[B]One-tailed Mann-Whitney test comparing experimental condition to each respective control. N/A, not applicable; Sig, significance; ****, p-value <0.0001

Table S19: *EMI1*$^{+/-}$ clones have significant increases in 53BP1 signal intensities relative to NT-Control.

Condition	n[A]	53BP1 Total Signal Intensity/Cell (×10^5)		Sig[C]
		Mean	SD[B]	
NT-Control (DMSO)	404	1.554	0.4759	N/A
NT-Control +Bleomycin	405	2.450	1.409	****
NT-Control	352	1.537	0.4382	N/A
EMI1$^{+/-}$1	389	1.749	0.8545	****
EMI1$^{+/-}$2	446	2.026	1.130	****

[A]n, number of interphase cells analyzed

[B]SD, standard deviation

[C]One-tailed Student's t-test comparing experimental condition to each respective control. N/A, not applicable; Sig, significance; ****, p-value <0.0001

Table S20. Multiple T-tests reveal significant changes in proliferation rates from early to late passages in *EMI1*$^{+/-}$ clones.

Passage	Condition	n[A]	Doubling time (h)	p-value[B]	Sig[C]
p0	NT-Control	6	20.5	N/A	N/A
	EMI1$^{+/-}$1	6	20.4	0.7992	ns
	EMI1$^{+/-}$2	6	23.8	0.0595	ns
p20	NT-Control	6	21.6	N/A	N/A
	EMI1$^{+/-}$1	6	16.9	0.0002	***
	EMI1$^{+/-}$2	6	17.7	0.0019	**

[A]Number of wells analyzed
[B]p-values calculated from the multiple t-tests for the listed condition at day 6 (144 h) post-seeding relative to NT-control; N/A, not applicable
[C]Sig, Significance; ns, not significant, p-value > 0.05; **, p-value < 0.01; ***, p-value < 0.001

Table S21. Welch's T-tests identify significant increases in *EMI1*$^{+/-}$ colony numbers over time.

Passage	Condition	n[A]	Mean Colony Number	p-value[B]	Sig[C]
p0	NT-Control	2	2.5	N/A	N/A
	EMI1$^{+/-}$1	2	2.5	>0.9999	ns
	EMI1$^{+/-}$2	2	2	0.8109	ns
p20	NT-Control	2	11.5	N/A	N/A
	EMI1$^{+/-}$1	2	62	0.0331	*
	EMI1$^{+/-}$2	2	53	0.0040	*

[A]Number of wells analyzed
[B]p-values calculated from Welch's t-test relative to NT-control; N/A, not applicable
[C]Sig, Significance; ns, not significant, p-value > 0.05; *, p-value < 0.05

Table S22. Welch's T-tests uncover significant changes in *EMI1*$^{+/-}$ colony sizes over time.

Passage	Condition	n[A]	Mean Colony Size (mm^2)	p-value[B]	Sig[C]
p0	NT-Control	2	0.012	N/A	N/A
	EMI1$^{+/-}$1	2	0.011	0.1521	ns
	EMI1$^{+/-}$2	2	0.011	0.2042	ns
p20	NT-Control	2	0.012	N/A	N/A
	EMI1$^{+/-}$1	2	0.011	0.6101	ns
	EMI1$^{+/-}$2	2	0.019	< 0.0001	****

[A]Number of wells analyzed
[B]p-values calculated from Welch's t-test relative to NT-control; N/A, not applicable
[C]Sig, Significance; ns, not significant, p-value > 0.05; ****, p-value < 0.0001
